# Treatment of post-transplant recurrent FSGS in children using plasmapheresis and augmentation of immunosuppression

**DOI:** 10.1186/s12882-022-02768-w

**Published:** 2022-04-05

**Authors:** Jaime M. Restrepo, Laura Torres-Canchala, Hernando Londoño, Eliana Manzi, Michael J. G. Somers

**Affiliations:** 1 Pediatric Nephrology and Transplantation, Fundación Valle del Lili, Cali, Colombia; 2 Sister Renal Center Program, International Society of Nephrology, Brussels, Belgium; 3 Outreach Program, International Pediatric Transplant Association, Mount Laurel, USA; 4 Facultad de ciencias de la salud , Universidad Icesi, Cali, Colombia; 5 Centro de Investigaciones Clínicas, Fundación Valle del Lili, Cali, Colombia; 6 Division of Nephrology, Boston Children’s Hospital, Boston, MA USA

**Keywords:** Focal and segmental glomerulosclerosis, Renal transplantation, Recurrence, Plasmapheresis, Cyclophosphamide, Pediatric

## Abstract

**Background:**

Up to 60% of pediatric renal transplant recipients with end-stage renal disease due to primary focal and segmental glomerulosclerosis (FSGS) may develop recurrent disease. Such recurrence is associated with poor prognosis if no remission is achieved. We report a single center experience with a protocol based on plasmapheresis and increased immunosuppression that resulted in a high long-lived remission rate.

**Methods:**

This retrospective cohort study included consecutive pediatric renal transplant patients with recurrent FSGS treated with a standardized protocol using plasmapheresis and cyclophosphamide to supplement usual post-transplant immunosuppression with calcineurin inhibitors and steroids. Relapse was defined as urinary protein/creatinine ratio > 1.0 g/g and remission as < 0.5 g/g.

**Results:**

Seventeen patients with FSGS recurrence post-transplant were treated. All had therapy resistant FSGS in native kidneys and had been on dialysis from 4 to 10 years. Of the 17, one died perioperatively from a pulmonary thromboembolism. Fifteen others achieved a complete remission within 3 months of treatment for FSGS recurrence. After a median follow-up period of 4 years, there were no recurrences of significant proteinuria. One patient achieved remission with rituximab.

**Conclusion:**

The addition of plasmapheresis and cyclophosphamide to a calcineurin- and steroid-based immunosuppression regime was highly successful in inducing high remission rates with recurrent FSGS. Prospective trials are needed to evaluate further the efficacy of increased immunosuppression along with plasmapheresis in this setting.

## Introduction

While renal transplantation is the treatment of choice for children with end-stage renal disease (ESRD) [1], some diseases such as primary focal and segmental glomerulosclerosis (FSGS) recur in the allograft and adversely affect graft outcomes if no remission is achieved [2–5]. Recurrence rate with FSGS as high as 60.5% have been reported, and concern for recurrence is widespread when considering renal transplantation in children with FSGS [6]. Although a large number of therapies have been employed to treat FSGS recurrence, there is currently no consensus treatment [2, 7] and most of the treatments have not been evidence-based.

Historically, plasmapheresis (PLEX) is the most widely used treatment modality for recurrent FSGS in both children and adults [2, 4]. One meta-analysis comprised of a single large series of 432 patients as well as several smaller case studies found a partial or complete remission rate of 71% with PLEX [8]. There may have been substantial reporting bias in this cohort, however, and the number of PLEX treatments varied sIgnificantly, with poor definition of other potentially beneficial concurrent therapies.

Similarly, the reports of PLEX efficacy in pediatric patients has varied. A recent publication in Pediatric Transplantation reported a limited impact of PLEX in treating FSGS recurrence [9], but on the other hand, a study of recurrent FSGS in Israeli children demonstrated a 78% partial or complete response, similar to the meta-analysis results [10]. There has also been interest coupling PLEX with Rituxumab or using LDL-apheresis rather than conventional plasma exchange, though reports of these approaches have also been limited [11–13].

Another approach for the treatment of FSGS has focused on increased immunosuppression along with PLEX [5]. Nathanson reported that 6/14 patients with FSGS recurrence achieved complete remission by using a combination of high dose cyclosporine, PLEX, and oral cyclophosphamide [5]. To discern if there were similar efficacy, we were interested in evaluating our experience with a similar protocol that adds cyclophosphamide to calcineurin inhibitors, steroids, and PLEX for post-transplant FSGS recurrence in children and adolescents.

## Methods

### Setting and study population

This study was performed at Fundación Valle del Lili Hospital in Cali, Colombia, a tertiary care university institution. Our long-standing pediatric renal transplant program keeps a local registry of all renal transplant patients at the institution. In our retrospective analysis, we included all patients < 18 years of age transplanted between 1998 and 2015 due to primary FSGS who manifested FSGS recurrence post-transplant and were treated with a protocol for FSGS recurrence based on the use of PLEX and addition of cyclophosphamide to baseline immunosuppression with a calcineurin inhibitor and steroids. This study was performed in accordance with the Declaration of Helsinki and was approved by the Fundación Valle del Lili ethics board committee. Informed written consent was waived by the Fundación Valle del Lili ethics board committee (“Comité de ética en Investigación Biomédica” in Spanish) since it was a retrospective cohort study without influencing patient care.

### Definitions, anthropometrics, and laboratory measurements

Recurrence was defined as a serum albumin level below 3 g/dL, either in the setting of a urinary protein/creatinine ratio (UPC) > 1 g/g presenting in the first 24 to 72 h after transplantation or the later onset of UPC > 1 g/g post-transplant distinct from other events such as acute rejection, acute kidney injury, or chronic allograft nephropathy, all documented by biopsy. Remission was defined as UPC < 0.5 g/g and serum albumin level > 3 g/dL.

Height was measured without shoes on a wall mounted stadiometer and weight was measured without heavy clothes using either a digital or balance-beam scale. Body mass index (BMI) was calculated as weight (kg)/height (m2). We calculated age- and gender- independent height and weight z-scores as previously described, using the Ped(z) app with the WHO reference intervals [14].

Blood samples were collected in anticoagulant and lithium heparin tubes. Creatinine was isotope dilution-mass spectrometry traceable and measured using a calorimetric modified Jaffe method and alkaline picrate [15]. Estimated glomerular filtration rate (eGFR) was calculated from the average of the three height measurements and serum creatinine with the new modified Schwartz formula [16].

### Peri- and post-transplant management

- All living donor (LD) recipients received 3 pre-emptive PLEX treatments during the week prior to transplantation and IV cyclosporine at 3.5 mg/Kg/day over the 24 h prior to transplant.
- Induction therapy was chosen at the discretion of the transplant surgeon or the transplant nephrologist and varied by era of transplantation. Five patients (29.4%) received thymoglobulin at a dose of 1–1.5 mg/kg/dose for a total of 4 daily doses maximum. Twelve patients (70.5%) received a monoclonal anti IL-2 receptor antibody (basiliximab or daclizumab). Basiliximab was given at a dose of 20 mg in children < 20 Kg (*n* = 8 [80%]) or 30 mg in larger children, with the first dose on the day of transplant and the second dose on day 4. Daclizumab (*n* = 7 [41.1%]) was provided at a dose of 1 mg/kg on the day of transplant and a second similar dose 2 weeks later.
- Steroid therapy: IV Prednisolone (10 mg/kg) daily for 3 days followed by oral prednisone tapering (1 mg/kg/day) over 2 months to a maintenance dose of 0.2 mg/kg/day for 2 years. Steroids were then tapered to alternate day for one more year and then discontinued.
- Calcineurin inhibitor therapy: High dose microemulsified cyclosporine A was prescribed at 8–10 mg/kg/day every 12 h, targeting a C2 level of 1200–1500 mcg/L, for 3 months. If the patients had no recurrence, they were then transitioned from cyclosporine to tacrolimus at a dose of 0.1–0.2 mg/kg/day. Trough target levels of tacrolimus were 8–10 μg/L for 6 months and then 6–8 μg/L.
- Mycophenolate mofetil (MMF) at 600–900 mg/m2/day. No MMF monitoring was employed.
- Cytomegalovirus Prophylaxis: Since 2008, all patients received daily oral valganciclovir prophylaxis (7 mg/m2 x creatinine clearance [16]) for cytomegalovirus prophylaxis for a total of 6 months.

### Treatment protocol for FSGS recurrence

(g) Upon diagnosis of recurrence, PLEX was initiated with 4 daily sessions, followed by every other day sessions as long as UPC > 0.5. If after 15 PLEX sessions there was no significant improvement in proteinuria, PLEX was discontinued. PLEX treatments consisted of 1.5 plasma volume exchange swith 5% albumin as replacement fluid through a central vascular access using a Cobe Spectra System.
(h) Cyclophosphamide: Oral cyclophosphamide was prescribed at 2 mg /kg/day daily once daily for 3 months in all patients who had FSGS recurrence, regardless of the cumulative dose of cyclophosphamide that may have been provided prior to transplantation to treat native FSGS. When cyclophosphamide was started, MMF was held. When cyclophosphamide was completed, MMF was re-started at 600–900 mg/m2/day.
(i) Calcineurin inhibitors: As detailed above, microemulsified cyclosporine A at a dose of 8–10 mg/kg/day in two divided doses was provided, targeting a C2 level of 1200–1500 mcg/L, for 3 months. If remission were achieved, patients were then converted after 3 months to tacrolimus at a dose of 0.1–0.2 mg/kg/day, targeting through levels of 8–10 μg/L during the first year, and then 6–8 μg/L thereafter.
(j) Rituximab: In one child who had persistent leukopenia during cyclophosphamide therapy, a single dose of rituximab 375 mg/m2 was provided when the cyclophosphamide therapy needed to be stopped prior to its full provision. In a second patient with partial remission after PLEX and completion of cyclophosphamide, rituximab was also provided, with four weekly doses of 375 mg/m2.

### Statistical methods

Anthropometric z-scores were calculated using the app Ped(z) based on WHO growth charts [14]. We used simple descriptive statistics wherever possible. Continuous variables were analyzed for normal distribution using the D’Agostino-Pearson normality test. No adjustments were made for missing data. All calculations were performed using GraphPad Prism version 5.0f for Mac or STATA 14.0 for PC registered to LTC.

## Results

Nineteen children with end-stage renal failure due to primary FSGS received their first kidney transplant at Fundación Valle del Lili and were part of the local transplant registry. Seventeen (89.4%) experienced recurrence of FSGS and were treated according to the protocol outlined above. Table 1 presents demographic and clinical management characteristics of transplanted children with recurrence.

**Table 1:** FSGS Patient demographic and clinical characteristics

Characteristics	***N*** = 17
**Male,*n*(%)**	11 (64.7)
**Age at trasplant, years. Median (IQR)**	14.3 (10.8–17.2)
**Time between diagnosis and transplant, years. Median (IQR)**	5.8 (3.9–7.4)
**Donant type, *n*(%)**	
Live donor	10 (58.8)
Deceased donor	7 (41.2)
**Antropometrical measurements,**	
Weight [Kg], median (IQR)	34 (26–48)
Weight z-score, mean (±SD)	−1.1 (1.2)
Height [cm], median (IQR)	150 (124–155)
Height z-score, mean (±SD)	−1.8 (0.9)
BMI [Kg/m2], median (IQR)	18.3 (15.2–19.3)
BMI z-score, mean (±SD)	−0.7 (1.1)
**Previous nephrectomy,*n*(%)**	10 (58.8)
**Pre-transplant Plasmapheresis,*n*(%)**	8 (47.1)
**Pre-transplant dialysis modality,*n*(%)**	
Hemodialysis	10 (58.8)
Peritoneal dialysis,	8 (47.1)
**Induction therapy,*n*(%)**	
Thymoglobulin	5 (29.4)
Basiliximab	5 (29.4)
Daclizumab	7 (41.2)
**Patients Undergoing Plasmapheresis,*n*(%)**	
Plasmapheresis procedures, median (IQR)	9 (6–11)
**Remission with therapy,*n*(%)**	
Live donor	5/7
Deceased donor	7/10
**Acute Rejection,*n*(%)**	3 (17.7)
**Injert Lost,*n*(%)**	8 (35.3)
**Length of hospital stay (days), median (IQR)**	28 (19–46)
**Death,*n*(%)**	5 (29.4)

Eleven patients (64.7%) were boys. The median age at diagnosis of primary FSGS was 8 years (IQR 6.1–10.3). None of the patients was HIV positive, diabetic, or obese, or had any other cause of secondary FSGS. Genetic studies were not performed prior to transplantation in any child.

Prior to ESRD diagnosis, 14 of the 17 patients manifested steroid-resistance, with one child showing partial responsiveness. Prior to ESRD, all children had also been treated with non-steroid immunosuppression trying to induce FSGS remission, including 7 who received cyclosporine, 17 who received cyclophosphamide, and 2 who received Rituximab.

All children reached ESRD and required dialysis prior to transplantation (range 4–10 years). Ten patients (58%) required nephrectomy prior to transplant due to ongoing nephrosis or severe proteinuria. The median age at transplant was 14.3 years (IQR 10.8–17.2). Ten patients (58.8%) had a living donor graft and 7 (41.2%) a deceased donor graft. There were no differences in patient characteristics between children who received living related and deceased donor transplants.

In the 17 patients with recurrent FSGS who received post-transplant PLEX and augmented immunosuppression per protocol, the median number of PLEX procedures provided was 9 (IQR 6–11). Complete remission was achieved by 15 patients after starting PLEX and augmenting immunosuppression, with an additional patient achieved remission with the addition of rituximab after not responding to the course of cyclophosphamide and PLEX. One patient died of a pulmonary thromboembolism within 2 weeks of transplant while receiving PLEX therapy for recurrence in a still-functioning graft.

Nine patients had cellular and perihiliar variants two patients had no otherwise specified three patients had three patient had cellular variant and three had perihiliar variant [16].

Table 2 details quantification of proteinuria longitudinally. All patients who achieved remission stayed in remission without further disease recurrence during follow-up (median 4 years; IQR 2.2–7.8 years). In the single patient who did not remit, proteinuria transiently exacerbated further about 3 months after transplant concomitant with medication non-adherence, but then returned to prior levels.

**Table 2:** Longitudinal Proteinuria

Protein/Creatinine ratio, median (IQR)	***n*** = 17
At diagnosis of recurrence	6.2 (3.0–10.1)
At hospital discharge	0.35 (0.25–0.66)
3 months post-transplant	0.3 (0.25–0.4)
12 months post-transplant	0.095 (0.08–0.10)
72 months post-transplant	0.1 (0.095–0.16)

Table 3 details graft function by estimated GFR. Median estimated GFR stayed stable during the first year after transplant. At last follow-up at a median of 4 years after transplant, there had been a median decline of eGFR by 15 ml/min/1.73 M2.

**Table 3:** Longitudinal Renal Function

Renal function	***n*** = 17
**At 7 days, median (IQR)**	
Serum creatinine [mg/dL]	1.63 (0.58–2.89)
Schwartz eGFR [mL/min/1.73 m^2^]	50.6 (27.5–84.6)
**At 15 days, median (IQR)**	
Serum creatinine [mg/dL]	1.1 (0.7–2.8)
Schwartz eGFR [mL/min/1.73 m^2^]	61.2 (27.1–78.8)
**At 30 days, median (IQR)**	
Serum creatinine [mg/dL]	1.0 (0.8–2.9)
Schwartz eGFR [mL/min/1.73 m^2^]	60.3 (28.6–67.9)
**At discharge, median (IQR)**	
Serum creatinine [mg/dL]	1.0 (0.8–1.7)
Schwartz eGFR [mL/min/1.73 m^2^]	61.0 (42.2–74.0)
**At year, median (IQR)**	
Serum creatinine [mg/dL],	1.04 (0.78–2.3)
Schwartz eGFR [mL/min/1.73 m^2^]	57.7 (30.7–77.1)
**At last follow-up, median (IQR)**	
Time of follow-up	4.0 (2.2–7.8)
Serum creatinine [mg/dL]	1.63 (1.2–2.3)
Schwartz eGFR [mL/min/1.73 m^2^]	42.8 (33.1–55.8)
**Protein/Creatinine ratio, median (IQR)**	
At diagnosis of recurrence	6.2 (3.0–10.1)
At hospital discharge	0.35 (0.25–0.66)
3 months post-transplant	0.3 (0.25–0.4)
12 months post-transplant	0.095 (0.08–0.10)
72 months post-transplant	0.1 (0.095–0.16)

Regarding post-transplant infections, 2 (12%) had bacteremia due to *Staphylococcus aureus*, 2 (12%) 2 had bacteremia due to Kleibsella pneumoniae and 1 (6%) due to Acinetobacter baumanii. There were no cases of BKV polyoma infection, herpes virus infection or urinary tract infection. All patients received antibiotic prophylaxis during the first month after transplant.

In the child with persistent nephrosis, graft function was lost 64 months after transplant. Three other grafts were also lost during long-term follow-up: one related to inability to obtain immunosuppression and the others related to chronic allograft nephropathy. None of these patients had significant proteinuria at the time of graft loss. All patients who lost grafts subsequently died while on dialysis, two of infection, one from sequelae of a hypertensive crisis, and one as a young adult of colon cancer after having had another kidney transplant that also failed due to FSGS recurrence.

## Discussion

In this single center retrospective review of 17 children transplanted due to FSGS, 15 or 88% had recurrent disease and were treated for their recurrence with a protocol using PLEX and augmentation of immunosuppression. Of the children with recurrence, 15 or 88% were able to enter a long-lived complete remission. In all but one case, the remissions occurred quickly after institution of therapy for recurrence, and only one child required supplemental therapy with the addition of rituximab after not fully remitting after PLEX and cyclophosphamide provision.

Although a large meta-analysis of patients with FSGS recurrence demonstrated a similar 71% rate of response with PLEX-based therapies, nearly 30% achieved only a partial remission, and the number of PLEX sessions and the duration of PLEX therapy was often substantial. In comparison, the children described in our report tended to have rapid and complete responses, and there were no subsequent relapses during long-term follow up. Moreover, eGFR remained acceptable long-term, with eGFR decline over time like rates seen more generally in children.

There is currently no consensus about the best overall approach for the treatment of recurrent FSGS in children [2, 6, 7], largely due to the lack of prospective randomized controlled trials. Moreover, although PLEX is quite often used as a first-line therapy for recurrence, it is almost always provided in the setting of other additional therapies, and there is little data to guide clinicians as to the specific efficacy of individual components of such multi-tiered management [17].

The decision to augment immunosuppression in FSGS recurrence in children is further complicated since most of these children have already had significant pre-transplant exposure to immunosuppression as part of attempts to reverse FSGS in their native kidneys. Concerns for cumulative toxicity of certain medications, such as cyclophosphamide, may lead to decisions to forgo the use of what may be an efficacious therapy, especially when there is not clarity as to the best overall therapy or the best population for that drug’s efficacy. In the patient with colorectal cancer, the time between the use of cyclophosphamide and the appearance of colorectal cancer was 3 years. A cause-effect relationship cannot be confirmed or ruled out.

The paucity of prospective controlled studies not only exacerbates the lack of clarity as to best current management strategies but also complicates the assessment of novel therapies as they become available. For instance, rituximab and LDL-apheresis have both been used increasingly to treat FSGS recurrence, but their utility in children with recurrent disease in general is difficult to assess when there has been little formal assessment of current therapies to allow baseline levels of expectation.

The importance of identifying the best therapies is underscored by long-term outcomes in successfully transplanted patients as compared to those with failed allografts. FSGS recurrence that does not respond to treatment leads to early graft loss and return to dialysis, and most often pretends future episodes of recurrence with subsequent transplantation. The long-term reliance on dialysis for renal replacement therapy adversely affects quality of life, requires more health care resources and is more costly, and results in significantly shorter life expectancy. In our own cohort, despite the successful treatment of recurrence in almost all patients, those who went on to lose graft function for other reasons and then required chronic dialysis as young adults all had limited life spans.

This study has some limitations. First, among patients with nephrotic syndrome, there is a low incidence of FSGS that leads to chronic kidney failure and requires a transplant, which explains the low number of patients presented in this study [18]. Second, due to the lengthy collection time, the patients were subjected to different types of management and different clinical concept, which may lead to information bias. However, this study has strengths. The study was conducted in a tertiary care center. Long-term follow-up was possible in these patients from their nephrotic syndrome diagnosis.

Although the conclusions that can be drawn from our results may not be generalizable to all children with recurrent FSGS, our report does show that in some children an aggressive approach to recurrence that uses PLEX and immunosuppression augmentation including cyclophosphamide can lead to rapid, complete, long-term remission. Rigorous assessment of proposed protocols and their effectiveness in children with FSGS should help to determine what therapies need to be studied in a randomized, controlled fashion in children and underscore the importance of sharing results such as described with this reported cohort to the pediatric nephrology community at large.

## Conclusion

The addition of plasmapheresis and cyclophosphamide to a calcineurin- and steroid-based immunosuppression regime was highly successful in inducing high remission rates with recurrent FSGS. Prospective trials are needed to evaluate further the efficacy of increased immunosuppression along with plasmapheresis in this setting.

## Data Availability

The datasets generated and/or analysed during the current study are not publicly available to protect patient information but are available from the corresponding author on reasonable request.
